# Ellipsoidal patellar bone tunnel fixation with Toggleloc suspension system for medial patellofemoral ligament reconstruction: A 5 years follow-up

**DOI:** 10.1097/MD.0000000000038379

**Published:** 2024-06-28

**Authors:** Uğur Özdemir, Bekir Murat Çinar, Mehmet Türker, Ahmet Çağri Uyar, Muhammed Fatih Serttaş, Abdülhalim Akar, Erhan Şükür, Alauddin Kochai

**Affiliations:** a Consultant Orthopaedic Surgeon. Department of Orthopedics and Traumatology, Bandırma Onyedi Eylül University Faculty of Medicine, Balıkesir, Turkey; b Consultant Orthopaedic Surgeon. Department of Orthopedics and Traumatology, Sakarya University Faculty of Medicine, Sakarya, Turkey; c Consultant Orthopaedic Surgeon. Department of Orthopedics and Traumatology, Kocaeli City Hospital, Kocaeli, Turkey; d Consultant Orthopaedic Surgeon. Department of Orthopedics and Traumatology, Emsey Hospital Hastanesi, İstanbul, Turkey.

**Keywords:** endobutton, MPFL reconstruction, patella dislocation, patellar instability

## Abstract

**Background:**

This study aimed to evaluate the clinical and radiological features of the patella fixation technique using Toggleloc suspension system in a single ellipsoidal blind patellar tunnel during medial patellofemoral ligament (MPFL) reconstruction.

**Methods:**

This study included 52 patients (25 men, 27 women) who underwent MPFL reconstruction using a semitendinosus tendon graft. The graft was fixed to the ellipsoidal single blind tunnel opened on the medial side of the patella with an endobutton and was fixed to the femoral tunnel by using bioabsorbable screw. Clinical scores (Kujala score, Lysholm score, Tegner activity score and the visual analog scale [VAS] score) were evaluated preoperatively and at the end-follow up. Preoperative and postoperative radiological measurements (trochlea depth, sulcus angle, patellar height, patellar congruence angle, patellar tilt angle and lateral patellofemoral angle) were evaluated with X-ray (Merchant X-ray, anteroposterior and lateral radiography) and computed tomography (CT) of the knee.

**Results:**

Postoperative patellar redislocation or subluxation was not observed in any patient. Patellar congruence angle, patellar tilt angle and lateral patellofemoral angle mean values were found to return to normal values in the postoperative period and the results were statistically significant. Also statistically significant improvement in all clinical scores postoperatively. According to the Insall-Salvati index (ISI) and Caton-Deschamps index (CDI) on lateral radiography of the knee at 30° flexion, patellar height decreased in the postoperative period statistically significant. The CDI was above 1.3 in 17 (%32) of our patients. Thirteen of these values decreased to normal values. No radiological progression of patellofemoral osteoarthritis was observed in all patients at the final follow-up evaluation.

**Conclusion:**

In cases of patellofemoral instability, fixation of the tendon graft in blind ellipsoid tunnel using the Toggleloc suspension system provides satisfactory patellar graft fixation strength, significant functional improvement and a low failure rate.

## 1. Introduction

Recurrent patellar dislocation is a common knee disorder and occurs after multifactorial causes including soft tissue imbalance and bone pathoanatomy. Patellofemoral joint stability is provided by the harmonious operation of static and dynamic stabilizers as well as bone structure and lower extremity alignment. Recent anatomical and biomechanical studies have highlighted the complexity of the patellofemoral joint and medial stabilizers of the patella.^[1–3]^ Nowadays, the most researched popular anatomical structure among the causes of patellofemoral dislocation is the medial patellofemoral ligament (MPFL).

As the MPFL is the primary stabilizer of the patella, it contributes 60% of the medial forces resisting lateral displacement of the patella in the early knee flexion (0°–30°).^[1–3]^ Therefore, up to 96% of patients suffer damage in cases of the lateral patella instability. In these cases, the rate of redislocation after primary patellar dislocation was treated without surgery was estimated to be 30% to 70%.^[4]^ Accordingly, several authors have suggested that repair or reconstruction of the MPFL may be advocated to reduce the high incidence of recurrence.^[5–8]^ However, MPFL reconstruction is recommended in cases of recurrent patellar instability due to insufficient healing capacity of the ligament and increased laxity in the medial patellar retinacular structures and very successful surgical results are obtained.

In MPFL reconstruction; graft option, configuration and patellar fixation method are extremely important in obtaining the effect of native MPFL on patellofemoral biomechanics. Numerous repair techniques using a variety of transplants and fixation methods have been described. Several techniques were introduced to fix the graft to the patellar MPFL attachment site, including the patellar bone tunnel and suture anchor techniques.^[9–11]^ The fact that each method has its own advantages and disadvantages has led to the fact that a standardized reconstruction method has not yet been defined. It has been reported in the literature that especially if the graft is fixed in the tunnel in the patella, it threatens fractures and increases the risk of dislocation when fixed with suture fixation.^[12,13]^

Our hypothesis was that ellipsoidal bone tunnel tendon fixation will reduce the risk of redislocation provides better ligament healing and more anatomic graft positioning. This study aimed to evaluate the clinical and radiological results of the patellar fixation technique using Toggleloc™ with Ziploop™ (Zimmer Biomet, Indiana) technology device in a single ellipsoidal blind patellar tunnel during MPFL reconstruction.

## 2. Method

**Figure 1.**
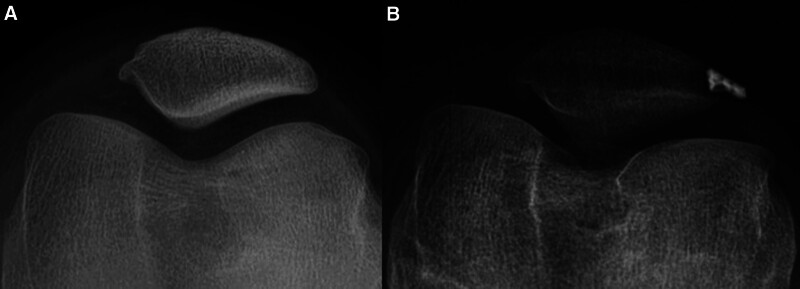
Preoperative (A) and postoperative (B) Merchant graphics of the patient who underwent MPFL reconstruction. MPFL = medial patellofemoral ligament.

**Figure 2.**
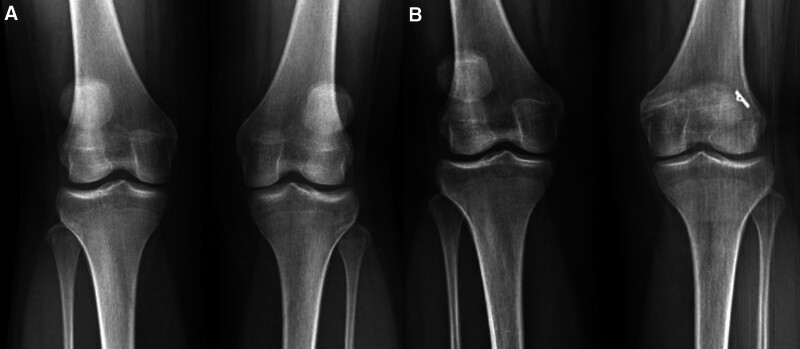
Preoperative (A) and postoperative (B) anterior-posterior X-ray graphics of the patient who underwent MPFL reconstruction. MPFL = medial patellofemoral ligament.

**Figure 3.**
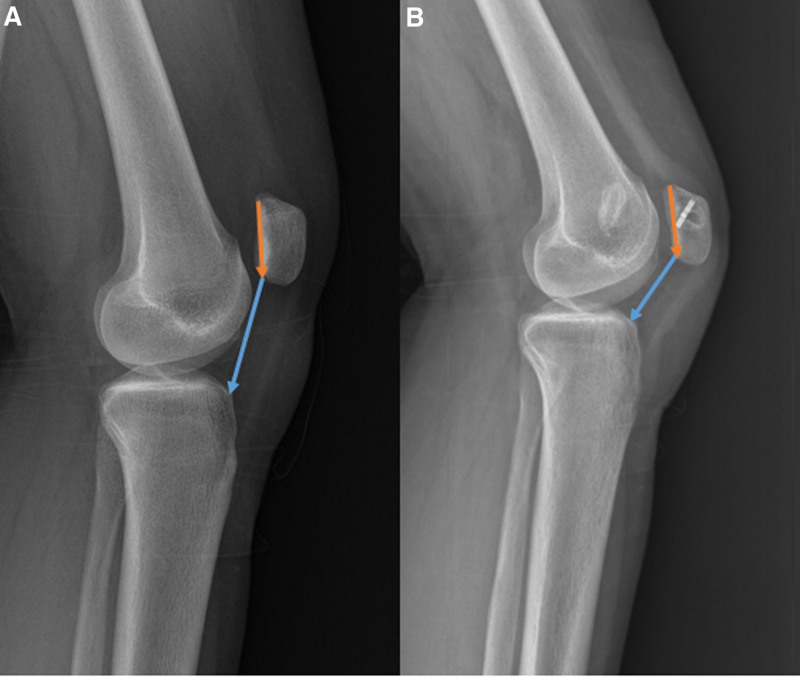
Preoperative (A) and postoperative (B) lateral radiographs of the knee at 30° flexion. The radiographs show patellar height indices. Caton-Deschamps index ratio of the distance between the anterosuperior point of the tibial plateau and distal pole of the patella (blue line) to the joint patellar surface (orange line).

After obtaining institutional review board approval (02/05/2023, E-71522473-050.01.04-241700-146), a retrospective study was conducted from 2015 to 2022. MPFL reconstruction was performed in 52 patients (27 female, 25 male) due to patellofemoral instability. Patients aged 15 to 50 years, who were diagnosed with MPFL rupture on magnetic resonance imaging (MRI), had a history of at least 2 patellar dislocations, or whose instability symptoms (pain and/or subluxation) continued despite appropriate physical therapy and rehabilitation after the first dislocation were included in this study. Patients with a history of recurrent patellar dislocation but with multiple ligament injury, trochlear dysplasia, lower extremity malalignment and/or rotational deformity in the long bone, and patients who had previously undergone ipsilateral knee surgery were excluded from the study. Each patient was examined by a single physician for the patellar apprehension test, amount of lateral translation, abnormality in patellar tracking (positive “‘J-sign’”), and degree of knee flexion at which instability occurred, and preoperative and postoperative values were recorded. All patients had radiographs (Merchant X-ray [Fig. 1], standing AP [Fig. 2], and lateral radiography at 30° flexion), computed tomography (CT) and MRI in the preoperative period and at the first postoperative follow-up. In Merchant graphy; sulcus angle and patellar congruence angle were measured. The lateral knee radiography at 30° flexion was performed to analyze patellar height that was quantified using the ISI and Caton-Deschamps index (CDI) method was used (Fig. 3). In CT imaging of the knee at 30° flexion; patellar tilt angle and lateral patellofemoral angle were measured. MPFL rupture and cartilage lesions of the patellofemoral joint were evaluated with MRI. Clinical scores (Kujala score, Lysholm score, Tegner activity score and VAS score) were evaluated preoperatively and at the end-follow up. Patients were followed up at 1, 3, and 6 months postoperatively, and then every 6 months at least 1 year.

### 2.1. Surgical technique

**Figure 4.**
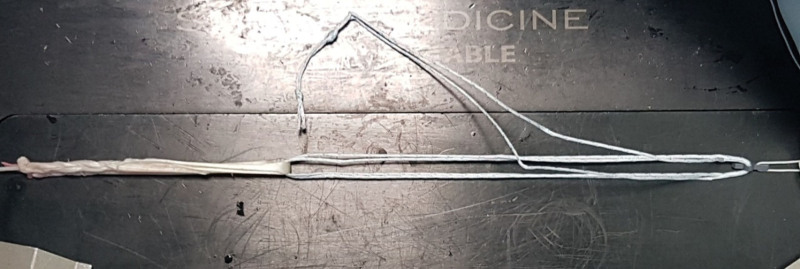
Semitendinosus autograft placed in a “Loop” form in the suspensory button fixation system.

**Figure 5.**
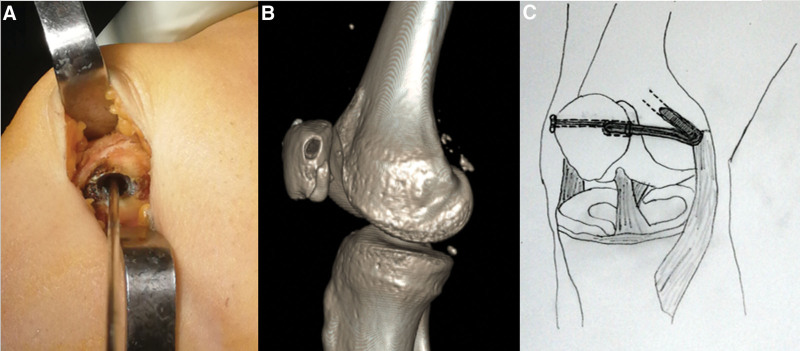
(A) Intraoperative visuals of the ellipsoid-shaped blind patellar tunnel. (B) Postoperative 3D-CT imaging of ellipsoid-shaped blind patellar tunnel (C) drawing of the ellipsoidal patellar tunnel and MPFL reconstruction technique we applied. MPFL = medial patellofemoral ligament.

**Figure 6.**
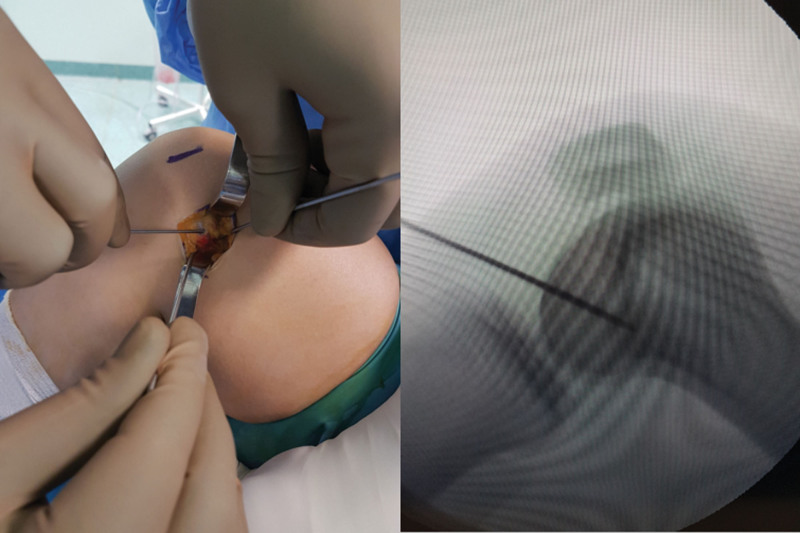
Determination of the MPFL femoral attachment site under fluoroscopy as described by Schöttle. MPFL = medial patellofemoral ligament.

**Figure 7.**
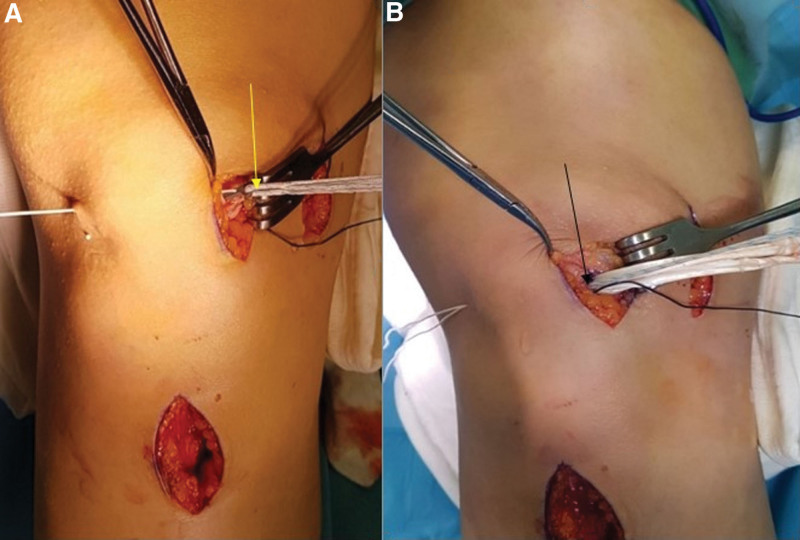
(A) After patellar tunnel opening, image of semitendinosus tendon graft placed in a “loop” form in the Toggleloc suspensory button fixation system (yellow arrow) connected to a guide rope passed in mediolateral. (B) Image of the “loop” graft placed in the ellipsoid blind patellar tunnel (black arrow) opening on the medial edge of the patella.

All surgical procedures were performed by a single surgeon. Arthroscopic examination was always performed before reconstruction surgery to detect associated injuries of the joint. Once this was completed, an anteromedial approach was performed to the proximal third of the tibia for semitendinosus muscle tendon graft harvesting. The ipsilateral semitendinosus tendon was used as autograft for all patients. After the muscle tissues were cleaned from the tendon, the 2 free ends of the tendon, which were placed in the suspensory button fixation system in the form of a “loop,” were stitched together. The length and the thickness of the tendon graft was measured (Fig. 4). Then, a longitudinal incision of approximately 2 cm was made on the superomedial edge of the patella to reach the area where the MPFL attaches to the patella. From this point, a 2.4 mm guide wire was sent transversely under fluoroscopy so as not to damage the chondral surfaces and anterior cortex. By drilling over this guide wire with a cannulated 4.5 mm drill, a tunnel was opened through which the suspensory button fixation system could pass. The guide wire was removed and the entrance part of the tunnel was widened using a burr drill according to the thickness of the loop-shaped tendon graft. Thus, an ellipsoid-shaped blind tunnel was obtained in the superomedial edge of the patella (Fig. 5). Afterwards, a longitudinal incision of approximately 4 cm was made on the medial side of the knee, at the point where the adductor tubercle and medial epicondyle were palpated. The skin and subcutaneous tissues were dissected without damaging the medial infrapatellar branch of the saphenous nerve. The radiographic method described by Schöttle^[14]^ was used to accurately determine the femoral insertion site of the MPFL (Fig. 6). The 2.4 mm guide wire was directed proximally and slightly anteriorly from this point and take out from the lateral edge of the femur. Using a cannulated drill of appropriate diameter, drilling was performed over this wire and a blind femoral tunnel was obtained, which was 1 mm wider than the thickness of the tendon graft we prepared. Afterwards, a soft tissue space was created between both tunnels by subretinacular blunt dissection which the tendon graft could pass. The carrier ropes of the suspensory button fixation system were passed through the patellar tunnel with the help of a guide wire and taken to the lateral side. By pulling these ropes from the lateral side, the button was passed through the tunnel and placed on the lateral edge of the patella longitudinally. Then the graft, which was folded in a “loop” shape, was placed firmly in the ellipsoid-shaped patellar blind tunnel by pulling the free ropes of the suspensory system. (Fig. 7) The part of the graft remaining outside the patellar tunnel was pulled through the subretinacular soft tissue space toward the femoral tunnel. Then, the ropes of the free graft ends were passed through the femoral tunnel with the help of a guide wire and taken to the lateral edge of the femur. After the free tendon ends were placed in the femoral tunnel by pulling the ropes, the knee was flexed to 20° to 30° and the graft was fixed using an absorbable screw at a tension that would allow half patella width movement. Post-reconstruction arthroscopic examination revealed normal patellar excursion during knee flexion and extension movements.

### 2.2. Postoperative rehabilitation

After MPFL reconstruction, the same rehabilitation program was followed in all patients. On the first postoperative day, isometric quadriceps contraction and range of motion (ROM) improving exercises were started. By using double crutches and a locked knee brace in extension, the patients were allowed to ambulation with the weightbearing they could tolerate. The load was gradually increased and full load was started at the end of the 2nd week. After the 2nd week, straight leg raise exercise were started in patients whose inflammation findings had regressed, who had achieved 90 degrees of knee flexion and who could walk safely with full weight bearing on crutches. At the end of the 6th week, patients who achieved full knee flexion, had good quadriceps control (straight leg raise without extensor lag and able to stand single leg on the operated side) were discontinued using knee braces and crutches after gaining functional ROM, muscle strength and stability, mild sports activity was started in the 3rd postoperative month. At the end of the 6th month, patients with improved balance and proprioception and good neuromuscular control were allowed to return to their normal sports activities.

### 2.3. Statistical analysis

Mean, standard deviation, median, minimum and maximum values were reported for numerical variables while frequency (n) and percentages (%) were given for categorical ones. Normality assumption was tested via Kolmogorov-Smirnov test. Student *t* test was applied to compare 2 independent groups, and Wilcoxon Test was performed to assess the dependent pre-op and post-op measures. Spearman correlation coefficients were provided to evaluate the relationship between preop-postop measures and other parameters in dataset. SPSS 21.0 IBM Corp. Released 2012. IBM SPSS Statistics for Windows, Version 21.0. Armonk, NY: IBM Corp. was used for all analyses. Statistical significance was taken as *P* ≤ .05 in all analyses.

## 3. Results

The mean age at surgery was 23.9 years (range 15–47 years). The mean postoperative follow-up duration was 64.8 months (range 12–84 months). The mean duration of complaints of patients before surgery was 15.9 months (range 1–48 months). The patient demographics and characteristics are shown in Table 1. Patellar instability developed at an average of 31.82 ± 6.08° of flexion in the preoperative examination performed under anesthesia. Postoperative patellar redislocation or subluxation was not observed in any patient. While preoperative apprehension test was positive in all patients, positivity continued in 8 (%15) postoperative patients, even though there was no instability. Basic preoperative and postoperative radiological features are summarized in Table 2. The mean depth of the trochlea was 5.65 ± 0.94 mm, and the mean sulcus angle was 138.48 ± 3.16 degrees. According to the ISI, the patellar height ratio was 1.03 ± 0.18 in the preoperative period and 0.94 ± 0.12 in the postoperative period (*P* < .001). At the same time, the preoperative CDI was 1.3 ± 0.21 and decreased in the postoperative period (1.15 ± 0.15) (*P* < .001). The CDI was above 1.3 in 17 (%32) of our patients. Thirteen of these values decreased to normal values. Patellar height, congruence angle and tilt angle, lateral patellofemoral angle mean values returned to normal values in the postoperative period and the results were statistically significant (*P* < .001). No radiological progression of patellofemoral osteoarthritis was seen in any case at the final follow-up evaluation. At the final follow-up, there was a statistically significant improvement in the Kujala scores between the pre- and postoperatively (from 55.84 to 84.38; *P* < .001). Similarly, the overall functional score improvements were observed in Tegner and Lysholm scores, respectively (*P* < .001). The basic preoperative and postoperative clinical features are summarized as follows: Table 3. In addition, the mean VAS score values of the patients decreased from 6 ± 1.72 to 1.07 ± 0.98 (*P* < .001). This statistically significant decrease indicates that there is a significant regression in the patient complained of anterior knee pain during the preoperative period. According to the Crosby-Insall satisfaction evaluation system, the patient satisfaction rates were 9.6% good and 90.4% very good. Seven of our patients had a positive J-sign before surgery; this finding was not found in the follow-up of these patients.

**Table 1:** Patient demographics and characteristics.

Number of patients	52
Gender (female/male)	27/25
Side (left/right)	28/24
Mean age (yr)	23.9 ± 9.36
Age range (yr)	15–47
Mean follow-up (mo)	64.8 ± 15.9
Follow-up range (mo)	12–84
Mean complaint period (mo)	15.9 ± 10
Complaint period range (mo)	1–48
Apprehension test positivity (preoperative/postoperative)	52/8

**Table 2:** Preoperative and postoperative radiological values.

Radiological measurement	Preoperative	Postoperative	*P* value
Trochlea depth (mm)	5.65 ± 0.94	-	-
Sulcus angle	138.48 ± 3.16	-	-
Patellar height (ISI)	1.03 ± 0.18	0.94 ± 0.12	<.001
Patellar height (CDI)	1.3 ± 0.21	1.15 ± 0.15	<.001
Patellar congruence angle	18.53 ± 6.29	0.82 ± 3.28	<.001
Patellar tilt angle	22.01 ± 5.27	12.38 ± 3.79	<.001
Lateral patellofemoral angle	0.96 ± 3.46	16.67 ± 5.84	<.001

CDI = Caton-Deschamps index, ISI = Insall-Salvati index.

**Table 3:** Preoperative and postoperative clinical score values.

Functional scores	Preoperative	Postoperative	*P* value
Tegner score	3.80 ± 0.99	6.90 ± 1.62	<.001
Lysholm score	53.73 ± 12.43	88.15 ± 8.31	<.001
Kujala score	55.84 ± 8.55	84.38 ± 7.73	<.001
VAS score	6 ± 1.72	1.07 ± 0.98	<.001
Crosby-Insall Satisfaction Scala: Bad (0) - %0 Good (5) - %9,6 well(47) - %90.4			

VAS = the visual analog scale.

In our study, complications were observed in 2 patients. Patellar fractures developed in 1 patient (%1.9) during the follow-up period. The patient, a professional boxer, was allowed to start mild sports activities in the 3rd postoperative month. However, during training, due to the patient squatting exercise underweight, a transverse type of patella fracture developed and surgical treatment was performed for the fracture. Joint mobilization was performed under anesthesia in 1 patient (%1.9) due to flexion limitation (flexion limited to 90°) in the first month of surgery. At the final follow-up, 115° knee flexion was achieved. In all other patients, the full ROM was achieved.

## 4. Discussion

Since the femoral fixation method is more standardized than the patellar fixation in MPFL reconstruction surgery, different patellar fixation methods have been described in the literature such as suture anchor, interference screw fixation of the graft, and docking techniques.^[6, 9, 11, 15, 16]^ The result analyzes of similar studies in the literature were summarized as follows: Table 4. Successful results have been reported with in-tunnel or anchor-assisted graft surface fixation methods, which are frequently used today, the optimal method has not yet been determined. In-tunnel fixation methods provide greater biomechanical strength and an advantage in terms of tendon-bone healing. The most important aspect of graft bone healing is the surface area where they come into contact with each other. Therefore, there may be a difference in intra-tunnel screw-assisted fixation and suspension methods owing to the gathering of the graft in a region within the tunnel. Another important point is that the patella fixation method and graft configuration become clinically, anatomically and biomechanically controversial due to the wide fan attachment of the ligament to the patella. Therefore, it may be necessary to open a double tunnel in the patella to reconstruct the double-bundle ligament in order to obtain a functional anatomical ligament. Although stronger biomechanical results have been obtained with tendon graft fixation in the tunnel, which is a disadvantage in that may occur as patellar fractures.^[13, 17]^ The risk of clinical fracture; depends on the number of tunnels, their positions and diameters, respectively. In particular, double tunneling may increase the risk of patellar fractures in cases. Hopper et al reported an anterior patellar stress fracture incidence of 5.6% with the use of the transverse patellar tunnel technique.^[18]^ In order to avoid the risk of fracture, Papp and Cosgarea described the “docking” technique, which provides patellar fixation with a transverse blind tunnel.^[19]^ Lenshow et al reported that the risk of fracture was very high in the “bone bridge” and transverse double-tunnel techniques that applied biomechanical tests, and no fractures occurred in the “docking” technique. This is because, unlike the transverse tunnel technique, docking technique, a blind tunnel was opened at a depth of approximately 20 mm, rather than along the entire patella.^[20]^ In studies where this technique is applied, a circular entry site 6 mm in diameter is often required to inserted a semitendinosus tendon graft into the tunnel. If there is a thin patella, it may weaken of the patella. By applying our technique on the medial side of the patella, we created an ellipsoidal tunnel entrance by keeping the width smaller but the length longer, thus obtaining a wide tendon-bone contact surface without weakening of the patella. We also used this technique to reduce the risk of fracture. However, only 1 patient developed a patella fracture in this study.

**Table 4:** The result analyzes of similar studies in the literature.

References	Technique (number of knees)	Results
Heo et al^[6]^ (Meta-analysis)	Group 1: Suture anchor fixation Group 2: Double transpatellar tunnel fixation	The mean patellar redislocation rates in these 2 groups were 3.4% and 3.2% (*P* = .879). The mean improvements in the Kujala score in these 2 groups were 37.2 and 28.7 (*P* = .018). The mean improvements in the Lysholm score in these 2 groups were 41.0 and 35.9 (*P* = .547)
Sim et al^[11]^ (Clinical)	Patellar fixation by using suture anchor in double-bundle configuration and femoral fixation by using the adjustable-length loop device (12).	No patients experienced surgical complications, including patellar fracture and redislocation. The mean Lysholm score improved from 71.7 ± 3.2 to 93.3 ± 5.6 (*P* < .001). The mean Kujala score improved from 67.3 ± 8.8 to 90.3 ± 5.7 (*P* < .001). The mean pVAS score from 4.7 ± 1.2 to 1.3 ± 1.1 (*P* < .001).
Kalinterakis et al^[12]^ (Review)	Group 1: Patellar fixation with implant Group 2: Patellar fixation without implant	Postoperative Kujala (*P* = .89) and Lysholm (*P* = .26) scores were better in the implant-free techniques. A higher rate of recurrent dislocation (*P* = .4), subluxation (*P* = .019) and stiffness (*P* = .55) was noted in the implant-free techniques. A higher rate of patella fractures (*P* = .09), reoperation (*P* = .17) and infection (*P* = .33) was noted in the implant-based techniques
Astur et al^[15]^ (Clinical)	Group 1: Endobutton (30) Group 2: Suture Anchor (28)	Group 1: Patella fracture (1), knee discomfort (3) and arthrofibrosis (1) Group 2: Arthrofibrosis (1) There was no statistical difference among postoperative Kujala, Fulkerson, and SF-36 questionnaires scores between Groups 1 and 2 in patients evaluated 2 and 5 years after surgery. There was a slight tendency for better surgical results in Group 1 technique
Mohammed et al^[16]^ (Clinical)	Group S: Single patellar tunnel - Endobutton (29) Group D: Double patellar tunnel - Interference screw fixation (29)	Group S: İnstability (2), anterior knee pain (6), knee discomfort (3) Group D: Anterior knee pain (3)
Weinberger et al^[17]^ (Review)	Graft configuration (double vs single) Graft source (autograft vs allograft)	Double-limbed reconstructions were associated with both improved postoperative Kujala scores and a lower failure rate. Postoperative failure, as defined by recurrent patellar instability, favored double-limbed configurations (5.5 vs 10.6 %, *P* = .03). Autograft reconstructions were associated with greater postoperative improvements in Kujala scores when compared to allograft (32.2 ± 2.5 vs 22.5 ± 2.0, *P* < .001)
Hopper et al^[18]^ (Clinical)	Interference screw fixation in a bony tunnel (68)	5.6% postoperative patellar fractures (4). Tight reconstruction requiring further release (1). Recurrent dislocations (12). High failure rate was seen in patients with high-grade trochlear dysplasia.
Lenschow et al^[20]^ (Biomechanical)	Group 1: Interference screws Group 2: Transpatellar tunnels technique. Group 3: Suture anchor Group 4: Bone bridge technique. Group 5: Transosseous suture technique	The transosseous suture technique had significantly less stiffness techniques (*P* < .05). The highest load to failure was seen in the transosseous suture technique (*P* > .05)
Hapa et al^[21]^ (Biomechanical)	Group A: Transverse tunnel technique. Group B: Interference screws Group C: Docking technique. Group D: Suture anchor	The docking group had lower stiffness technique (*P* = .007). The highest load to failure was seen in the transverse tunnel technique (*P* > .05) There was also no significant difference in the ultimate load between the anchor [299 (SD 116) N], tunnel [304 (SD 140) N], and interference screw groups [241 (SD 103) N]
Bartsch et al^[22]^ (Review)	Analyzed 6 studies describing outcomes after isolated medial patellofemoral ligament reconstruction with regard to patellar height.	Both patients with patella alta and normal patella height reported satisfactory outcomes after isolated medial patellofemoral ligament reconstruction.
Luceri et al^[23]^ (Clinical)	Relationship between isolated medial patellofemoral ligament reconstruction and patella height (95)	The CDI of 79.4% of the patella alta knees was reduced to within normal limits postoperatively. The CDI of 50% of the severe patella alta knees was reduced to within normal limits postoperatively.
Sappey-Marinier et al^[24]^ - (Clinical)	Suture Anchor (211)	%4.7 Recurrent dislocations (10). Analyses highlighted preoperative risk factors for failure as patella alta and preoperative positive J-sign
Shah et al^[25]^ - (Review)	Reviewed 25 articles describing MPFL reconstruction with clinical outcomes.	Patella fracture (4) occurred in 3 different studies used transpatellar tunnels. The suture techniques demonstrated a higher rate of recurrent dislocation/subluxation (4.8%) and apprehension/hypermobility (24.0%) than the tunnel technique (3.3% and 8.6%, respectively).

CDI = Caton-Deschamps index, MPFL = medial patellofemoral ligament, SD = standard deviation.

In the docking technique, graft-attached sutures are tied to the lateral side of the patella to complete the fixation; however, in our study, we used a Toggleloc™ with Ziploop™ technology device fixation material to provide more durable fixation. In addition, unlike previously described techniques, we created an ellipsoidal tunnel in the medial part of the patella and created a wider tendon-bone contact area. Lenshow reported that fixation failure in this technique was due to the untangling of threads, which they passed through the double tunnel and were tied together at the lateral edge of the patella. Hapa et al in their biomechanical study, they found the lowest ultimate load and stiffness values among the 4 different patellar fixation methods in the docking method.^[20, 21]^ Although this technique has a biomechanical weakness, in our Toggleloc™ with the Ziploop™ technology system, the “docking” technique do not have this disadvantage. However, an endobutton placed at the lateral edge of the patella may cause discomfort to patients. Astur et al in a study comparing “endobutton” and suture anchor fixations, both techniques produce patellar discomfort at the same rate.^[15]^ Mohammed et al in their studies, in which they used the “endobutton,” 3 patients reported discomfort on the lateral edge of the patella. Therefore, the material was removed, but we did not observe this complaint in any of the patients because we were seated on the lateral edge of the patella with a longitudinal course.^[16]^

Although there is currently no ideal scoring system for patellofemoral instability, we evaluated our patients using knee-specific questionnaires and we found satisfaction-enhancing evaluations of clinical values such as improvements in VAS scores and return to activities of daily living and sports. Similarly, we determined that patellar height, patellar congruence angle, patellar tilt angle and lateral patellofemoral angle values returned to normal by using the technique we applied. Some studies have highlighted some factors predicting the failure of this surgery in terms of clinical improvement and redislocation.^[17, 22–24]^ The most emphasized of these factors is the preoperative radiological patellar alta and clinically positive J-sign. In the literature, it has been emphasized that even with the application of MPFL reconstruction without a bone procedure, the negative effects of these factors can be reduced. Luceri et al found a radiological and clinical improvement in patients with mild patella alta (CDI 1.2–1.4) that did not require distal tibial tubercle transfer with the isolated MPFL reconstruction procedure they performed using the gracilis tendons in a double tunnel.^[23]^ Similarly, we determined a significant preoperative radiological patellar height value improvement in our patients by providing adequate medial support with the endobutton docking technique was applied. We believe that this is because of the biomechanical advantage provided by the endobutton suspension fixation system, which is different from the previously defined docking technique. However, we found apprehension test positivity without dislocation in 8 (15%) patients, especially with the patella alta at the last follow-up. Studies using tunnels or suture fixations techniques have reported a rate of 8.6% to 24%, respectively.^[25]^ We believe that the patellar fixation system used in our study may be due to the elongation of the system after a certain period. This situation similarly encountered in anterior cruciate ligament surgery.

Despite generally good results with MPFL reconstruction, some surgical aspects are still controversial. Astur et al reported a relationship between endobutton and suture fixation in MPFL reconstruction techniques, patients had similar results in their 2 to 5-year follow-up according to function and quality-of-life questionnaires, but emphasized that early-term results were better than late-term results in an endobutton system contrary to this view.^[15]^ But Mohammed et al showed increasing problems with single tunnel endobutton patellar fixation with more reoperation and failure rates compared to double-tunnel fixation.^[16]^ However, the weak point of both studies compared to ours is that they did not include radiological evaluation.

There is no consensus on the graft choice, configuration, and fixation methods, but there are extremely important in determining the effect of native MPFL on patellofemoral biomechanics. In a meta-analysis study investigating the effects of single or double-bundle repair on both clinical scores and redislocation rates in MPFL reconstruction surgery. Weinberger et al reported that the double-bundle graft configuration was superior to single bundle in reducing the rate of postoperative recurrent patellar instability and increasing the Kujala score improvement.^[17]^ However, Heo et al reported that suture anchor use of double leg graft or double transpatellar tunnel fixation did not significantly differ in patellar redislocation rates, and that clinical improvement was achieved with suture anchor fixation method, because biomechanical slack fixation reduces loading on the patellofemoral joint.^[6]^ Although graft fixation with a double patellar tunnel provides successful results and poses a risk in terms of the patella fracture. Therefore, to reduce this risk and obtain a wide tendon-bone contact surface, we applied graft fixation with docking technique in a single ellipsoidal tunnel, and we did not detect redislocation or anterior knee pain in any of our patients.

Our study has some inherent limitations because of its retrospective design. Furthermore, a lack of comparison with other techniques is a major limitation of the present study. The strengths of the study include: selecting the patients correctly, performing the operations by a fully equipped surgeon, continuing the follow-up of all patients, and having the follow-ups performed by the same surgeon. And also, a 5-year follow-up period and large sample size is sufficient to determine clinical outcomes, such as redislocation. Considering the significant increase in the clinical scores of the patients, the radiological normalization of patellar alignment parameters (patellar tilt angle, accommodation angle, lateral patellofemoral angle) and the significant regression in anterior knee pain complaints, this technique, which provides tendon graft fixation in the blind ellipsoid tunnel using Toggleloc with a Ziploop technology device, is a successful technique associated with good clinical and radiological outcomes. With this technique, the physiological kinematics and stability in MPFL reconstruction are provided anatomically and safely to avoid complications. However, more detailed data can be obtained through a prospective study comparing this technique with different reconstruction methods.

## 5. Conclusion

The results of the current study showed that, in cases of patellofemoral instability, fixation of the tendon graft in a blind ellipsoid tunnel (docking technique) using Toggleloc with Ziploop technology provided significant functional improvement and low failure rate. At the same time, it is shown that radiological parameters are improved as well as providing patient satisfaction.

## Author contributions

**Data curation:** Uğur Özdemir, Muhammed Fatih Serttaş.

**Formal analysis:** Uğur Özdemir, Abdülhalim Akar.

**Investigation:** Uğur Özdemir, Muhammed Fatih Serttaş, Abdülhalim Akar, Alauddin Kochai.

**Methodology:** Uğur Özdemir, Ahmet Çağri Uyar, Muhammed Fatih Serttaş, Alauddin Kochai.

**Project administration:** Uğur Özdemir, Erhan Şükür.

**Resources:** Uğur Özdemir, Ahmet Çağri Uyar, Erhan Şükür, Alauddin Kochai.

**Software:** Uğur Özdemir.

**Supervision:** Uğur Özdemir, Mehmet Türker, Alauddin Kochai.

**Validation:** Uğur Özdemir, Erhan Şükür, Alauddin Kochai.

**Visualization:** Uğur Özdemir, Erhan Şükür, Alauddin Kochai.

**Writing – original draft:** Uğur Özdemir.

**Writing – review & editing:** Uğur Özdemir, Bekir Murat Çinar, Mehmet Türker.
